# Digital Isolation: The Impact of Social Media and Emerging Technologies on Mental Health

**DOI:** 10.3390/healthcare14121701

**Published:** 2026-06-15

**Authors:** Mateusz Grajek, Teresa Wagner-Tomaszewska, Tomasz Jurys

**Affiliations:** 1 Department of Public Health, Faculty of Public Health, Medical University of Silesia in Katowice, 40-055 Katowice, Poland; 2 Department of Rehabilitation, Faculty of Health Sciences, Medical University of Silesia in Katowice, 40-055 Katowice, Poland

**Keywords:** digital isolation, social media, mental health, loneliness, FoMO

## Abstract

Digital isolation represents a contemporary paradox in which increased connectivity through social media and digital technologies does not necessarily translate into improved social integration or psychological well-being. This review synthesizes current evidence on the relationship between digital environments and mental health, with a focus on mechanisms underlying loneliness, anxiety, depression, and related outcomes. The findings indicate that problematic and passive use of social media—particularly when associated with social comparison processes and Fear of Missing Out (FoMO)—is consistently linked to increased levels of depressive symptoms, anxiety, sleep disturbances, and reduced well-being. At the same time, the evidence highlights substantial heterogeneity, suggesting that the impact of digital technologies is moderated by user characteristics, age, patterns of engagement, and psychosocial context. Importantly, digital technologies may also serve compensatory and protective functions by facilitating social support, especially in conditions of objective isolation. Key mediating mechanisms include cyberbullying, social exclusion, emotional contagion, and internalization of body image standards. The concept of “digital loneliness” emerges as a useful framework for understanding the discrepancy between constant connectivity and perceived relational insufficiency. Practical implications emphasize the need for targeted interventions focusing on digital literacy, healthy usage patterns, and psychosocial support rather than simplistic reduction in screen time. Overall, digital isolation should be conceptualized as a qualitative dysfunction of mediated social interaction rather than a purely quantitative effect of technology exposure.

## 1. Background

The concept of digital isolation describes one of the most characteristic paradoxes of modernity: an increase in the number of communication channels and the intensity of networked connections does not necessarily lead to greater closeness, belonging, or psychological support. On the contrary, in many studies, the digital environment appears as a space in which loneliness, anxiety, social comparison, cyberbullying, and feelings of inadequacy intensify—phenomena that are closely linked to mental health [1,2,3]. In this sense, digital isolation does not refer solely to being cut off from others, but also to the experience of relational emptiness amid constant communicative availability.

The provided sources situate the problem at the intersection of psychiatry, psychology, public health, cyberpsychology, education, and health informatics. On the one hand, they describe social media as a potential risk factor for depression, anxiety, sleep disturbances, body image problems, and suicidal behaviors; on the other, they show that technologies can also function as tools for compensating isolation, building support, and designing preventive and therapeutic interventions [4,5,6,7]. This duality should be preserved, as a simplified view of technology as unequivocally harmful does not reflect the actual state of the research.

Digital isolation can be conceptualized as a higher-order construct that integrates elements of perceived loneliness, relational dissatisfaction, and dysfunctions of mediated social interaction. Unlike traditional constructs such as loneliness or social isolation, which primarily refer to either subjective experience or objective social conditions, digital isolation reflects a discrepancy between technologically enabled connectivity and the perceived quality, authenticity, and emotional sufficiency of interpersonal relationships. From a theoretical perspective, this phenomenon can be situated at the intersection of several established frameworks. Social comparison theory provides a basis for understanding how continuous exposure to curated representations of others’ lives may intensify feelings of inadequacy and exclusion. Uses and gratifications theory highlights that the psychological outcomes of social media use depend on user motivations and patterns of engagement, distinguishing between active, socially oriented use and passive, consumption-based behaviors. Additionally, self-determination theory suggests that digital environments may differentially satisfy or frustrate basic psychological needs for relatedness, competence, and autonomy.

Importantly, the concept of digital loneliness should be understood as a theoretical and phenomenological construct rather than a formally recognized diagnostic category. It is used here as an interpretive framework describing the discrepancy between persistent digital connectivity and perceived relational insufficiency, rather than as a diagnosis included in DSM-5-TR or ICD-11.

Integrating these perspectives, digital isolation may be understood as a process in which specific modes of engagement with digital technologies interact with individual vulnerabilities and contextual factors, leading to adverse mental health outcomes. This framework allows for a more precise distinction between adaptive and maladaptive forms of technology use and provides a basis for interpreting heterogeneous findings in the literature.

The aim of the article is to review the available sources concerning the impact of social media and new technologies on mental health. The analysis seeks to address three main questions: first, what relationships between the digital environment and mental health most commonly emerge in the provided literature? Second, through which psychological and social mechanisms may technologies intensify or reduce the sense of isolation? Third, what practical implications follow from these studies for prevention, education, and clinical interventions?

## 2. Review Methods

This study was conducted as a scoping review following the methodological framework proposed by the Preferred Reporting Items for Systematic Reviews and Meta-Analyses extension for Scoping Reviews (PRISMA-ScR). The aim was to comprehensively map the existing literature on the relationship between social media use, digital technologies, and mental health outcomes, with particular emphasis on mechanisms of digital isolation, including loneliness, FoMO, and psychosocial mediators.

A scoping review approach was selected because the available literature is conceptually and methodologically heterogeneous, encompassing quantitative, qualitative, mixed-methods studies, systematic reviews, and meta-analyses. The primary objective was therefore to map the breadth of evidence, identify recurring mechanisms, and clarify conceptual relationships rather than to estimate pooled effect sizes.

A systematic search strategy was developed in consultation with the study objectives. Electronic databases including PubMed/MEDLINE, Scopus, Web of Science, and PsycINFO were searched for relevant publications. The search covered studies published between January 2010 and March 2026, reflecting the period of rapid expansion of social media platforms. Although the search covered publications from 2010 onward, studies meeting all eligibility criteria were identified only from 2021 onwards, reflecting the recent emergence of the digital isolation construct. Search strings combined controlled vocabulary (e.g., MeSH terms) and free-text keywords related to “social media”, “digital isolation”, “loneliness”, “mental health”, “depression”, “anxiety”, “FoMO”, and “cyberbullying”. Boolean operators (AND, OR) and truncation were applied to optimize sensitivity and specificity. Reference lists of included articles were additionally screened to identify further relevant studies. No review protocol was registered prior to conducting the study. Given the exploratory and mapping-oriented nature of the scoping review, protocol registration was not considered mandatory; however, eligibility criteria and screening procedures were defined a priori.

Eligibility criteria were defined a priori. Studies were included if they: (1) examined the relationship between social media or digital technology use and mental health outcomes; (2) addressed constructs related to isolation, loneliness, or psychosocial functioning; (3) were empirical studies (quantitative, qualitative, or mixed-methods), systematic reviews, or meta-analyses; and (4) were published in peer-reviewed journals in English. Exclusion criteria comprised: (1) studies not directly addressing mental health outcomes; (2) purely technical or engineering-focused publications without psychosocial analysis; (3) conference abstracts without full text; and (4) non-peer-reviewed sources.

The study selection process followed a two-stage screening procedure. First, titles and abstracts were independently screened by two reviewers to identify potentially relevant articles. In the second stage, full texts of eligible studies were assessed against the inclusion criteria. Discrepancies were resolved through discussion or consultation with a third reviewer. The initial database search yielded a total of 1284 records. After removal of duplicates (n = 312), 972 unique records remained for screening. Title and abstract screening led to the exclusion of 781 records that did not meet the inclusion criteria, primarily due to lack of direct relevance to mental health outcomes or absence of a clear focus on social media or digital technologies. A total of 191 full-text articles were assessed for eligibility. Of these, 172 studies were excluded for the following reasons: (1) insufficient focus on psychosocial or mental health variables (n = 71); (2) purely descriptive or technological scope without analytical linkage to digital isolation or well-being (n = 34); (3) lack of methodological transparency or incomplete data reporting (n = 27); (4) non-peer-reviewed or grey literature (n = 21); and (5) duplication of datasets or overlapping samples across publications (n = 19). Ultimately, 19 studies were included in the final synthesis.

**Figure 1:**
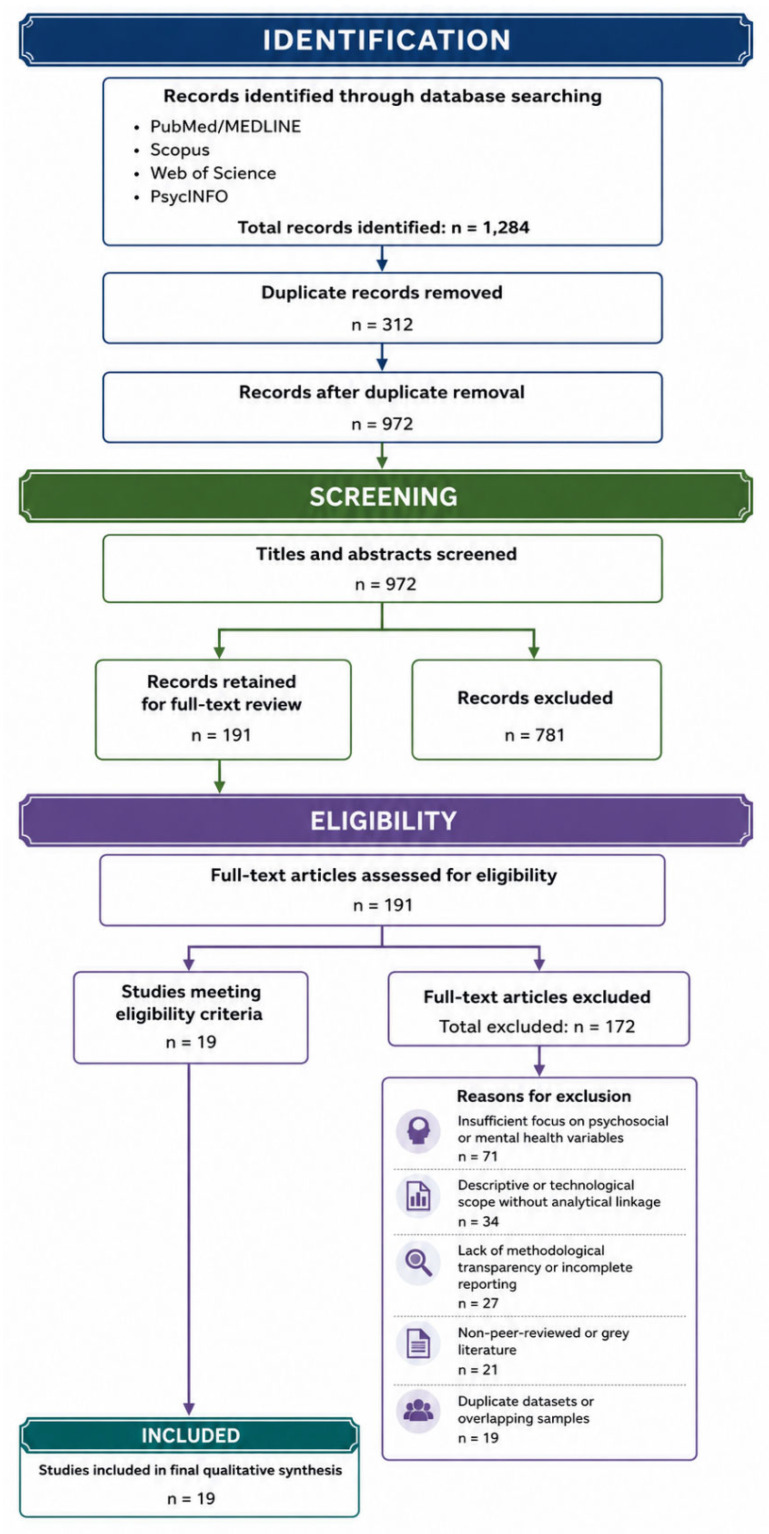
PRISMA-ScR flow diagram illustrating the identification, screening, eligibility assessment, and inclusion of studies investigating digital isolation, social media use, and mental health outcomes.

Study selection was based exclusively on predefined eligibility criteria. No consideration was given to authorship during screening or inclusion decisions. All eligible studies meeting the inclusion criteria were retained regardless of author affiliation. Data extraction was performed using a standardized charting form developed iteratively during the review process. Extracted variables included: author and year, study design, population characteristics (age, gender, sample size), type and intensity of social media use, measured mental health outcomes (e.g., depression, anxiety, loneliness), key mediating variables (e.g., FoMO, social comparison), and principal findings. Where applicable, contextual factors such as pandemic conditions, educational settings, or demographic differences were also recorded. Due to substantial heterogeneity in study designs, populations, outcomes, and operational definitions, findings were synthesized narratively. Evidence was grouped into three overarching domains: patterns of association, mediating psychological mechanisms, and population-specific moderators (Figure 1).

## 3. Review Results

Based on the reviewed evidence, a conceptual model of digital isolation can be proposed. In this model, patterns of digital engagement (e.g., passive use, nighttime use, high-frequency checking) function as primary predictors. These are mediated by psychological mechanisms such as Fear of Missing Out (FoMO), social comparison processes, cyberbullying exposure, and emotional contagion. The outcomes include depressive symptoms, anxiety, loneliness, reduced well-being, and sleep disturbances. Importantly, these relationships are moderated by individual and contextual variables, including age, gender, baseline mental health status, and the quality of offline social relationships. Such a model underscores that the effects of digital technologies are neither uniform nor deterministic, but contingent upon complex interactions between user characteristics and modes of engagement.

At the core of the issue lies the relationship between social isolation, loneliness, and the digital environment. Szawarnoga et al. [3] propose the concept of “digital loneliness” as a proposed conceptual framework in psychiatry and indicate that excessive use of social media is associated with increased feelings of loneliness, heightened anxiety, reduced sleep quality, lower self-esteem, and an overall deterioration in mental health. In their account, younger users, women, and individuals with disabilities are particularly vulnerable, while higher education is associated with less time spent online and greater awareness of potential risks. This perspective is complemented by Zhao et al. [8], who demonstrate that social isolation does not necessarily lead directly to addictive social media use but may operate indirectly through the phenomenon of Fear of Missing Out (FoMO; a psychological phenomenon involving feelings of anxiety or tension related to the belief that others are experiencing something valuable from which we are excluded) and a sequence linking social anxiety to FoMO. This suggests that the problem is not merely being alone, but also the psychological processing of relational deficits under conditions of constant visibility of others’ lives. It is important to distinguish between objective isolation and subjectively experienced loneliness. Shah and Househ [6] show that the distribution of reported loneliness among study participants follows a U-shaped pattern, particularly affecting younger and older individuals, while also highlighting the insufficient adaptation of technological interventions to the needs of younger groups. Research on older adults, in turn, suggests that the same technological tool may function differently depending on the user’s mental state: communication via social networking services may partially mitigate the effects of objective isolation, but does not necessarily reduce experienced loneliness and may sometimes even intensify it [7]. In this sense, digital isolation can be understood as a state of relational inefficacy of technology: channels of contact exist, yet they fail to restore a genuine sense of being with others.

A second key area addressed in current research concerns the relationships between social media use and psychological symptoms. Lopes et al. [4], in their systematic review, point to a strong and often bidirectional association between problematic social media use and depression or anxiety. At the same time, the authors emphasize that usage time alone does not explain the phenomenon: factors such as time of use (particularly nighttime use), emotional engagement, patterns of active versus passive participation, and gender differences are all significant. This is an important conclusion, as it shifts the focus from simple quantitative indicators to the qualitative aspects of digital experience. Ahmed et al. [1] reinforce this perspective in a meta-analysis focused on young people. They report small but statistically significant positive associations between social media use and both depression and anxiety, as well as a positive relationship between problematic use and depression, anxiety, and sleep disturbances, alongside a negative association with psychological well-being. The authors also highlight substantial heterogeneity in the findings, indicating that it is not methodologically sound to maintain the assumption of a uniform, identical effect of social media on all users. A more specific pattern is identified by Liu et al. [9], who found a linear relationship between time spent on social media and the risk of depressive symptoms among adolescents; each additional hour of use was associated with a 13% increase in depression risk, with a stronger effect observed in girls. The discrepancy between the conclusions of Liu et al. [9] and the more cautious position of Lopes et al. [4] does not represent a contradiction, but rather indicates that the significance of usage time depends on age, variable operationalization, and the population studied. A developmental perspective is added by Weigle and Shafi [10], who show that the impact of social media on young people’s mental health is complex and moderated by individual characteristics, such as susceptibility to social comparison or FoMO, as well as by specific experiences including cyberbullying or sexting. The authors also draw attention to the possibility of “emotional contagion” (the transfer of emotional states) within social media environments, while noting that most available evidence is correlational and based on self-report measures. Koh et al. [2], in turn, observe that 78.6% of the studies they reviewed reported negative effects of excessive and passive use, including increases in depression, anxiety, mood disturbances, and loneliness. However, approximately one-third of the studies also identified benefits associated with intentional and positive use, such as greater perceived social support and enjoyment. This suggests that the issue is not “social media” as a whole, but rather specific modes of engagement within its ecosystem.

The third identified issue related to social media use concerns cyberbullying, social exclusion, and suicidal risk. Ademiluyi et al. [11], in their systematic review, show that cyberbullying and social exclusion within social media are associated with depression and suicidal ideation, while also emphasizing that automated detection of cyberbullying remains insufficiently effective. Consequently, the best-supported standard at present remains social support and efforts aimed at restoring relationships, rather than relying solely on technical content filtering. The authors also note a shortage of longitudinal studies, which limits the ability to distinguish correlation from causation. Testoni et al. [12], in turn, indicate that the internet, social media, isolation, and loneliness constitute a significant theme in understanding suicide risk among adolescents and young adults. The protective measures they propose focus on institutional cooperation and the use of new technologies in prevention. This finding has theoretical implications: digital isolation emerges not only as an emotional state, but also as a factor requiring a systemic response.

The fourth area concerns body image and self-esteem. Laughter et al. [13] show that frequent exposure to social media can reinforce unrealistic appearance ideals, increase anxiety, and contribute to body dissatisfaction; among individuals with body dysmorphic disorder, it may intensify preoccupation with perceived flaws and encourage the pursuit of cosmetic or surgical procedures. Czubaj et al. [14] confirm the relevance of this mechanism among young people: exposure to fitspiration-type content was associated with decreased self-esteem in 37% of participants, particularly among women. Krupa-Kotara et al. [15] extend this finding, demonstrating that the internalization of body-related knowledge present in social media affects self-esteem and body image, although not uniformly across all groups. As a result, digital isolation may take the form of loneliness “exposed to evaluation”: the user remains constantly visible and subject to comparison, yet does not experience stable recognition or unconditional acceptance.

The fifth theme concerns age differences and the context of social media use. In a population-based study, Vazquez et al. [16] found that daily use of social media was associated with higher levels of loneliness among members of the baby boomer and silent generation cohorts, while no significant association was observed for millennials and Generation X. Svec et al. [7] demonstrate a similar complexity: among older adults, communication via social networking services may reduce stress related to objective isolation, but at the same time intensify negative affect in those who already feel lonely. Guzman et al. [17], in a systematic review focused on older adults, arrive at a comparable conclusion, emphasizing the importance of how use is conceptualized, as well as the structure of contacts, the nature of content, and the quality of interactions. Once again, this confirms that mere presence on social media has limited predictive value if the quality of relationships, mental health status, and broader life context are not taken into account.

A broader perspective on new technologies, extending beyond traditional social media, is provided by Melca et al. [18] and Geçer et al. [19]. In a study conducted during the COVID-19 pandemic, some participants used digital devices and social media as a substitute for direct sexual behaviors, which was interpreted as a strategy for reducing infection risk. This indicates that technologies can temporarily compensate for limitations in physical contact. At the same time, a qualitative study of academics on remote education shows that educational technologies offer flexibility, ease of connection, and in some cases, a reduction in social anxiety, but they also generate a sense of isolation, reduce motivation, and diminish the social interactivity of the learning process [19]. Digital isolation is therefore not solely a problem of social media platforms, but more broadly of a media-mediated mode of life. Plackett et al. [5], in a review of experimental studies, found that interventions based on therapeutic techniques, such as cognitive–behavioral approaches, were more likely to improve mental well-being than merely reducing social media use or complete abstinence. At the same time, the quality of these studies was low and the samples often non-representative. Shah and Househ [6] indicate that interactive technological solutions may be promising in reducing loneliness among young people, provided they are designed with their specific needs in mind rather than simply adapted from programs intended for older adults. Ademiluyi et al. [11] and Testoni et al. [12] consistently emphasize the importance of social support, cooperation between educational institutions and mental health services, and moving beyond purely technocratic solutions. Szawarnoga et al. [3] and Koh et al. [2], in turn, highlight the importance of education, risk awareness, and digital hygiene, without resorting to simplistic moralizing. The most well-supported practical model does not involve demonizing technology, but rather the careful development of competencies for its use.

Across the reviewed studies, several consistent patterns emerge despite methodological heterogeneity. First, the most robust associations are observed not for overall time spent on social media, but for specific qualitative patterns of use, particularly passive consumption, nighttime engagement, and behaviors driven by FoMO. Second, the relationship between social media use and mental health outcomes appears to be bidirectional in many cases, with pre-existing psychological vulnerabilities increasing susceptibility to problematic use, which in turn may exacerbate symptoms. At the same time, important inconsistencies remain. While some studies identify a linear dose–response relationship between time spent online and depressive symptoms, others suggest that this association weakens or disappears when controlling for mediating variables such as social comparison or emotional investment. This indicates that time-based metrics alone are insufficient as explanatory variables and should be interpreted within a broader psychosocial framework. A critical limitation of the current evidence base is the predominance of cross-sectional and self-report methodologies, which constrain causal inference and may inflate observed associations. The relative scarcity of longitudinal and experimental studies further limits the ability to determine the directionality and stability of observed effects over time.

## 4. Discussion

The findings of this review support the interpretation of digital isolation as a qualitatively distinct phenomenon that cannot be reduced to either objective social isolation or the subjective experience of loneliness alone. Instead, it reflects a dysfunction in the relational efficacy of digitally mediated interactions, where the presence of communication channels does not translate into meaningful social connection. One of the key contributions of the present analysis is the integration of heterogeneous findings into a process-oriented framework. The evidence suggests that the psychological impact of digital technologies is primarily driven by specific modes of engagement and mediating mechanisms, rather than by exposure per se. In particular, passive use, comparison-based interaction, and FoMO-related behaviors consistently emerge as central pathways linking digital environments to adverse mental health outcomes.

The reviewed literature also supports assumptions derived from Uses and Gratifications Theory, suggesting that mental health outcomes depend less on technology exposure itself and more on users’ motivations and patterns of engagement. Active, socially oriented use appears more likely to facilitate support and connectedness, whereas passive and comparison-based use is more consistently associated with adverse outcomes.

The observed heterogeneity across studies can be partially explained by differences in operationalization, population characteristics, and contextual factors. Developmental stage appears to be particularly relevant, with adolescents and young adults showing greater susceptibility to social comparison and peer-related pressures, while in older populations, the distinction between objective isolation and perceived loneliness becomes more salient. From a clinical and public health perspective, these findings challenge reductionist approaches that focus solely on limiting screen time. Instead, interventions should target the quality and structure of digital engagement, promote digital literacy, and address underlying psychosocial vulnerabilities. Therapeutic approaches, particularly those grounded in cognitive–behavioral frameworks, appear more promising than purely behavioral restrictions [1,2,3,4,5].

The scientific evidence presented in the review supports a coherent conclusion that digital isolation is primarily a problem of relationship quality and modes of participation in the digital environment, rather than merely a consequence of increased time spent online. The strongest and most consistently observed associations concern problematic, passive, FoMO-driven, and socially comparative patterns of social media use, which are linked to higher levels of depression, anxiety, loneliness, stress, sleep disturbances, and reduced well-being [6,7,8]. At the same time, the literature consistently emphasizes that technology can also serve a protective or compensatory function when it facilitates genuine support, active engagement, and high-quality interpersonal contact [11,13] (Table 1).

It is also important to emphasize that the effects are not uniform. Adolescents, young adults, women, and individuals particularly susceptible to social or appearance-related pressure appear to be at increased risk of depression, anxiety, body dissatisfaction, and digital loneliness. Among older adults, the key issue is the distinction between isolation and loneliness: social media may support those who are objectively isolated, but does not necessarily benefit those who are already deeply lonely, and may sometimes even intensify negative affect. From a practical perspective, the most well-supported strategies are those based on digital hygiene, education, social support, cyberbullying prevention, and therapeutic interventions that are better aligned with the mechanisms of problematic use, rather than simple “withdrawal” from social media.

Because most included studies were cross-sectional and relied on self-reported measures, the observed relationships should be interpreted primarily as associations. Although several mechanisms appear theoretically plausible, causal pathways remain insufficiently established and require confirmation in longitudinal and experimental research. Beyond summarizing existing evidence, the present review proposes an integrative framework in which digital isolation is conceptualized as an interaction between patterns of digital engagement, psychological mediators, and contextual moderators. This perspective may help reconcile previously inconsistent findings and provide a basis for future hypothesis-driven research.

## 5. Limitations

Several limitations of the present review should be acknowledged. First, as a scoping review, the study did not include a formal quality assessment of the included studies, which limits the ability to evaluate the strength of the evidence. Second, the reviewed literature is characterized by substantial methodological heterogeneity, including differences in study design, measurement tools, and operational definitions of both social media use and mental health outcomes. Third, the majority of studies rely on cross-sectional and self-report data, which restrict causal inference and may introduce reporting biases. The limited number of longitudinal and experimental studies further constrains the interpretation of temporal relationships and mechanisms. These methodological limitations reduce confidence in the strength and generalizability of the observed associations. Consequently, the conclusions of the present review should be interpreted as indicative of recurring patterns rather than definitive evidence of causal relationships. Additionally, publication bias cannot be excluded, as studies reporting significant associations may be overrepresented in the literature.

## 6. Conclusions

The available evidence indicates that digital isolation is primarily a function of the quality and structure of mediated social interactions rather than the amount of time spent online. The reviewed evidence suggests that digital isolation is more strongly related to the quality and structure of mediated social interactions than to the amount of time spent online. Problematic patterns of social media use—particularly passive engagement, FoMO-driven behaviors, and comparison-based interaction—are consistently associated with increased levels of depression, anxiety, loneliness, and reduced well-being. At the same time, digital technologies may serve protective and compensatory roles when they facilitate meaningful social connection and support. The effects of digital environments are therefore heterogeneous and moderated by individual characteristics, developmental stage, and psychosocial context. These findings underscore the need to move beyond reductionist, time-based approaches toward more nuanced models that account for mechanisms of use. Interventions should prioritize digital literacy, adaptive engagement patterns, and psychosocial support, rather than focusing solely on limiting exposure to technology.

## 7. Practical Implementation

From a practical perspective, the evidence supports a shift from quantitative to qualitative approaches in addressing the impact of digital technologies on mental health. Effective strategies should focus on promoting adaptive patterns of use, including active and socially meaningful engagement, as well as reducing behaviors associated with passive consumption and excessive social comparison. Interventions should incorporate digital literacy education, training in emotional regulation, and the development of critical awareness of online content. In clinical settings, integrating discussions of digital behavior into assessment and intervention protocols may enhance treatment effectiveness. At a systemic level, collaboration between educational institutions, mental health services, and technology platforms is essential to design environments that support psychological well-being rather than undermine it.

## Figures and Tables

**Table 1:** Identified problems associated with digital isolation and problematic social media use.

Issue	Domain	Mechanism	References
Digital loneliness and FoMO	distinction between isolation and loneliness, compensatory and mediating mechanisms	Cognitive–emotional mediation through FoMO and social anxiety; discrepancy between perceived and actual social connectedness; hypervisibility of others leading to internalized exclusion and relational dissatisfaction.	Szawarnoga et al. (2025) [3]; Zhao et al. (2025) [8]; Shah & Househ (2023) [6]; Vazquez et al. (2025) [16]; Svec et al. (2025) [7]
Problematic use and mental disorders	associations with psychological symptoms, intensity of use	Passive consumption and emotional overinvestment; upward social comparison; disrupted sleep (particularly nighttime use); bidirectional reinforcement between psychological vulnerability and maladaptive engagement patterns.	Lopes et al. (2022) [4]; Ahmed et al. (2024) [1]; Liu et al. (2022) [9]; Weigle & Shafi (2024) [10]; Koh et al. (2024) [2]
Cyberbullying, social exclusion, and suicide risk	online violence, exclusion, and marginalization	Exposure to online aggression and rejection; internalization of stigma; erosion of perceived social support; escalation of distress leading to depressive symptoms and suicidal ideation.	Ademiluyi et al. (2022) [11]; Testoni et al. (2021) [12]; Weigle & Shafi (2024) [10]
Body image and self-esteem	social comparisons, internalization of body norms	Internalization of idealized body standards; appearance-based social comparison; reinforcement of self-objectification; increased body dissatisfaction and reduced self-esteem.	Laughter et al. (2023) [13]; Czubaj et al. (2025) [14]; Krupa-Kotara et al. (2023) [15]
Differentiation of usage context	adolescents, young adults, older adults, pandemic, remote education	Moderation by developmental stage and psychosocial context; differential effects depending on baseline loneliness, type of interaction, and purpose of use; mismatch between technological affordances and user needs.	Liu et al. (2022) [9]; Shah & Househ (2023) [6]; Guzman et al. (2023) [17]; Vazquez et al. (2025) [16]; Svec et al. (2025) [7]; Melca et al. (2021) [18]; Geçer et al. (2023) [19]
Prevention and interventions	social support, therapeutic interventions, education and digital hygiene	Modification of cognitive and behavioral patterns (e.g., CBT mechanisms); strengthening perceived social support; development of digital literacy and self-regulation; shift from passive to active, meaningful engagement.	Plackett et al. (2023) [5]; Shah & Househ (2023) [6]; Ademiluyi et al. (2022) [11]; Testoni et al. (2021) [12]; Szawarnoga et al. (2025) [3]; Koh et al. (2024) [2]

## Data Availability

No new data were created or analyzed in this study.

## References

[1] Ahmed O., Walsh E.I., Dawel A., Alateeq K., Espinoza Oyarce D.A., Cherbuin N. (2024). Social media use, mental health and sleep: A systematic review with meta-analyses. J. Affect. Disord..

[2] Koh G.K., Ow Yong J.Q.Y., Lee A.R.Y.B., Ong B.S.Y., Yau C.E., Ho C.S.H., Goh Y.S. (2024). Social media use and its impact on adults’ mental health and well-being: A scoping review. Worldviews Evid.-Based Nurs..

[3] Szawarnoga D., Stasiniewicz A., Fojcik J., Krzystanek M. (2025). Digital loneliness as a new diagnostic category in psychiatry: The impact of technology and social media use on psychological well-being. Front. Psychiatry.

[4] Lopes L.S., Valentini J.P., Monteiro T.H., Costacurta M.C.F., Soares L.O.N., Telfar-Barnard L., Nunes P.V. (2022). Problematic social media use and its relationship with depression or anxiety: A systematic review. Cyberpsychology Behav. Soc. Netw..

[5] Plackett R., Blyth A., Schartau P. (2023). The impact of social media use interventions on mental well-being: Systematic review. J. Med. Internet Res..

[6] Shah H.A., Househ M. (2023). Understanding loneliness in younger people: Review of the opportunities and challenges for loneliness interventions. Interact. J. Med. Res..

[7] Svec J., Nemmers N., Lee J.E., Hwang I.J. (2025). Connected but lonely? The role of social networking sites among older adults experiencing isolation and loneliness. Aging Ment. Health.

[8] Zhao X., Xu T., Liu X. (2025). Social isolation and social media addiction: The serial mediation roles of social anxiety and FoMO among Chinese university students. Sci. Rep..

[9] Liu M., Kamper-DeMarco K.E., Zhang J., Xiao J., Dong D., Xue P. (2022). Time spent on social media and risk of depression in adolescents: A dose-response meta-analysis. Int. J. Environ. Res. Public Health.

[10] Weigle P.E., Shafi R.M.A. (2024). Social media and youth mental health. Curr. Psychiatry Rep..

[11] Ademiluyi A., Li C., Park A. (2022). Implications and preventions of cyberbullying and social exclusion in social media: Systematic review. JMIR Form. Res..

[12] Testoni I., Piol S., De Leo D. (2021). Suicide prevention: University students’ narratives on their reasons for living and for dying. Int. J. Environ. Res. Public Health.

[13] Laughter M.R., Anderson J.B., Maymone M.B.C., Kroumpouzos G. (2023). Psychology of aesthetics: Beauty, social media, and body dysmorphic disorder. Clin. Dermatol..

[14] Czubaj N., Szymańska M., Nowak B., Grajek M. (2025). The impact of social media on body image perception in young people. Nutrients.

[15] Krupa-Kotara K., Grajek M., Rozmiarek M., Malchrowicz-Mośko E., Staśkiewicz W., León-Guereño P., Aguirre-Betolaza A.M., Castañeda-Babarro A. (2023). The role of social media in internalizing body knowledge: A cross-sectional study among women with different food preferences. Int. J. Environ. Res. Public Health.

[16] Vazquez C.E., Falk D., Urbanski D.P., Kwong K., Abudu-Birresborn D., Bacsu J.D., Yoo-Jeong M., Chai H.W., Jung W., Smith M.L. (2025). The relationship between social media use and loneliness across the lifespan in the United States: Population-based study using Health Information National Trends Survey data. Adv. Patient Rep. Outcomes.

[17] Guzman A.A., Brecht M.L., Doering L.V., Macey P.M., Mentes J.C. (2023). Social media use and depression in older adults: A systematic review. Res. Gerontol. Nurs..

[18] Melca I.A., Nardi A.E., Gonçalves L.L., Ferreira R.M., de Padua M.S.K.L., King A.L.S. (2021). Sex, digital devices, social media, and social isolation: A study on sexual behavioral during COVID-19 pandemic. Clin. Pract. Epidemiol. Ment. Health.

[19] Geçer E., Bagci H., Atar C. (2023). “Nothing replaces meeting my students at class”: Analysing academics’ views regarding distance education. Educ. Inf. Technol..

